# A Unified Three‐Step Mirror‐Image Protocol for ECG, Echocardiography, and Cardioversion in Dextrocardia: A Case Report

**DOI:** 10.1002/ccr3.73122

**Published:** 2026-07-07

**Authors:** Junya Shimamoto, Rintaro Tamaruya

**Affiliations:** ^1^ Department of General Medicine Kokuho Ipponmatsu Hospital Ehime Japan; ^2^ Department of General Medicine Ehime Prefectural University of Health Sciences Ehime Japan; ^3^ Department of Internal Medicine Ehime Prefectural Minamiuwa Hospital Ehime Japan

**Keywords:** atrial fibrillation, cardioversion, dextrocardia, echocardiography, electrocardiography, situs inversus totalis

## Abstract

Dextrocardia with situs inversus totalis presents challenges in routine cardiovascular procedures. Although individual adaptations for electrocardiography, echocardiography, or cardioversion have been described, integrated guidance across these procedures remains limited. We report a man in his 50s with dextrocardia and situs inversus totalis who presented with atrial fibrillation and underwent electrical cardioversion. Electrocardiography, transthoracic echocardiography, and cardioversion pad placement were all performed using a mirror‐image configuration. Sinus rhythm was restored without complications. This case highlights that a unified mirror‐image approach across multiple procedures enables safe, reproducible management and provides a simple and reproducible framework for clinicians in emergency and primary care settings.

## Key Clinical Message

In dextrocardia, a mirror‐image approach facilitates cardiovascular management. ECG requires reversed limb leads and right‐sided precordial leads; echocardiography is optimized from the right hemithorax in the right lateral decubitus position; and cardioversion can be performed using mirror‐image pad placement across the right‐sided heart.

## Graphical abstract

A unified mirror‐image approach was successfully applied across electrocardiography, transthoracic echocardiography, and cardioversion in a patient with dextrocardia. Integrating these procedural adaptations into a single workflow may facilitate recognition and management of dextrocardia in emergency and primary care settings.

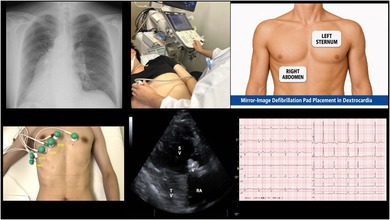

## 1 Introduction

Dextrocardia with situs inversus totalis is a rare congenital condition characterized by a mirror‐image arrangement of the thoracic and abdominal organs [1, 2]. Standard cardiovascular procedures, including electrocardiography, echocardiography, and cardioversion, are designed based on normal left‐sided anatomy and may be challenging to interpret or perform in such patients. Although procedural adaptations for each modality have been reported [3, 4, 5, 6, 7], they are typically described in isolation, and integrated guidance across multiple procedures remains limited. In clinical practice, however, clinicians encountering patients with dextrocardia may need to perform electrocardiography, echocardiography, and cardioversion during a single episode of care. Existing reports therefore require clinicians to integrate information from multiple sources in real time, which may be particularly challenging for primary care and emergency physicians who are unfamiliar with such anatomical variations. To our knowledge, no previous report has described a unified, clinically applicable mirror‐image approach integrating electrocardiography, echocardiography, and cardioversion within a single clinical workflow. We report a case in which a consistent mirror‐image approach was applied across electrocardiography, echocardiography, and electrical cardioversion, providing a simple and practical framework for clinical use.

## 2 Case History

**FIGURE 1:**
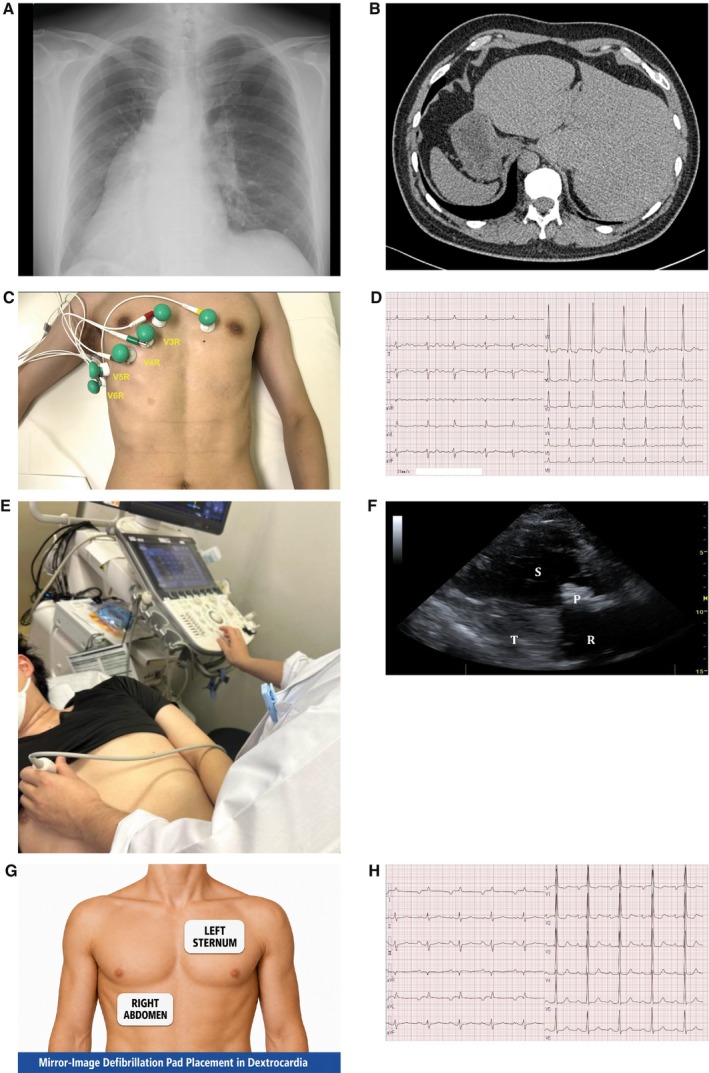
(A) Chest radiograph demonstrating mirror‐image thoracic anatomy consistent with situs inversus totalis. (B) Computed tomography demonstrating situs inversus totalis, with a complete mirror‐image arrangement of the thoracic and abdominal organs. (C) Mirror‐image ECG lead placement in dextrocardia showing reversed limb leads and right‐sided precordial leads (V1R–V6R). (D) ECG demonstrating atrial fibrillation with an irregular R–R interval and absence of discernible P waves. (E) Transthoracic echocardiography performed from the right hemithorax with the patient in the right lateral decubitus position, enabling adequate visualization of cardiac structures. (F) Transthoracic echocardiographic image obtained from the right hemithorax showing preserved systolic function of the dominant single ventricle (S), with visualization of the tricuspid valve (TV), pulmonary valve (PV), and right atrium (R), and no evidence of intracardiac thrombus. (G) Mirror‐image cardioversion pad placement in dextrocardia. One pad was positioned over the left upper parasternal region and the other over the right lower thoracoabdominal region to direct the shock vector across the right‐sided heart. (H) Electrocardiogram obtained after electrical cardioversion demonstrating restoration of sinus rhythm.

A man in his 50s with known situs inversus and dextrocardia, previously diagnosed at a tertiary care center and followed at a regional hospital, had a history of single atrium and functional single ventricle physiology (Figure 1A,B). He also had a history of atrial fibrillation treated with electrical cardioversion.

His baseline oxygen saturation was typically 82%–83%, and his resting heart rate ranged from the 50s to 60s. He presented with new‐onset dyspnea and palpitations, noting an increase in heart rate to approximately 88 beats per minute on the day of admission. He reported symptoms similar to those experienced during previous episodes of atrial fibrillation requiring cardioversion.

On presentation, blood pressure was 93/59 mmHg, heart rate was 62 beats per minute, and oxygen saturation was 84%.

## 3 Treatment

Given the presence of dextrocardia, precordial leads (V1–V6) were placed in a mirror‐image configuration on the right hemithorax, and limb leads were reversed accordingly (Figure 1C). Electrocardiography demonstrated atrial fibrillation with an irregular R–R interval and absence of discernible P waves (Figure 1D).

Transthoracic echocardiography was subsequently performed from the right hemithorax with the patient in the right lateral decubitus position, using mirror‐image probe positions summarized in Table 1; the examiner was seated on the patient's left side, with the ultrasound machine positioned accordingly (Figure 1E). This positioning was intentionally selected to optimize visualization of the right‐sided cardiac structures. Echocardiography confirmed a single atrium and functional single ventricle, identified the tricuspid and pulmonary valves with pulmonary valve doming consistent with stenosis, and demonstrated no evidence of intracardiac thrombus (Figure 1F).

**TABLE 1:** Mirror‐image adaptation of standard transthoracic echocardiographic views in dextrocardia.

View	Standard TTE	Mirror‐image TTE in dextrocardia	Index marker direction
Patient position	Left lateral decubitus	Right lateral decubitus	—
Parasternal long‐axis	Left parasternal border (3rd–4th intercostal spaces)	Right parasternal border (3rd–4th intercostal spaces)	Left shoulder
Parasternal short‐axis	Left parasternal border (3rd–4th intercostal spaces)	Right parasternal border (3rd–4th intercostal spaces)	Right shoulder
Apical four‐chamber	Left cardiac apex	Right cardiac apex	Right side
Subcostal	Subcostal window	Subcostal window	Right side
Suprasternal	Suprasternal notch	Suprasternal notch	Right shoulder

Based on these findings, recurrent atrial fibrillation without intracardiac thrombus was diagnosed. After thrombus exclusion, electrical cardioversion was performed using mirror‐image pad placement to encompass the right‐sided heart, with pads positioned over the left clavicular region and right lateral abdomen (Figure 1G). Sedation was achieved with midazolam (3 mg) and analgesia with pentazocine (3 mg). Continuous monitoring was performed during the procedure. A single synchronized shock of 100 J successfully restored sinus rhythm without complications (Figure 1H).

## 4 Discussion

This case suggests that a unified mirror‐image approach can be applied across multiple cardiovascular procedures in patients with dextrocardia. Although previous reports have described adaptations for individual procedures such as electrocardiography [3, 4], echocardiography [5], or cardioversion [6, 7] in patients with situs inversus and dextrocardia, these have generally been presented in isolation. A comparison with representative previous reports is summarized in Table 2. In contrast, our case integrates all three procedures within a single clinical pathway, demonstrating a coherent and practical strategy applicable in real‐world settings (Table 2). A key strength of this report is the integration of these procedure‐specific adaptations into a single, consistent clinical workflow.

**TABLE 2:** Comparison of previous reports and the present case.

Study	Focus of report	ECG	Echo‐cardiography	Cardioversion	Integrated workflow
Mozayan et al. [8]	ECG adaptation	✓	—	—	—
Lopez et al. [5]	Echocardiographic adaptation	—	✓	—	—
Gorenek et al. [7]	Cardioversion	—	—	✓	—
Cattermole et al. [6]	Defibrillation	—	—	Defibrillation only	—
Present case	Unified mirror‐image approach	✓	✓	✓	✓

Electrocardiographic assessment requires reverse modification of lead placement. As described in previous reports [8], precordial leads should be placed in a mirror‐image configuration on the right hemithorax. Without awareness of mirror‐image anatomy, standard lead placement may result in confusing or misleading findings, potentially delaying diagnosis or appropriate management [3]. Simple adjustments are particularly important in primary care and emergency settings, where electrocardiography frequently guides immediate clinical decision‐making.

Echocardiographic imaging poses additional technical challenges due to altered cardiac orientation. In this case, adequate visualization of cardiac structures was achieved by performing transthoracic echocardiography from the right hemithorax with the patient in the right lateral decubitus position. A mirror‐image approach was employed by adjusting probe positions and transducer orientations, as summarized in the practical protocol (Table 1) [9]. This approach enabled assessment of ventricular function and exclusion of intracardiac thrombus without the need for advanced imaging modalities. Familiarity with such positional and scanning adaptations may allow nonspecialist clinicians to obtain clinically useful information even in anatomically atypical patients.

Electrical cardioversion also requires thoughtful adaptation. In this case, cardioversion pads were applied in a right shoulder–left lower abdominal configuration, representing a mirror‐image of the standard anterolateral pad placement [10], to ensure that the cardioversion vector traversed the right‐sided cardiac mass. Previous reports in patients with dextrocardia have described anatomically adjusted anterolateral pad positioning, typically placing the sternal paddle in the right parasternal area and the apical paddle over the right‐sided ventricular apex [7]. Notably, successful defibrillation of ventricular fibrillation has also been reported using conventional anterolateral pad positions, suggesting that exact pad location may be less critical than prompt shock delivery in life‐threatening arrhythmias [6]. While anatomically optimized pad placement is desirable, restoration of circulation should not be delayed by uncertainty regarding exact positioning, particularly in urgent clinical situations.

From a primary care perspective, this case underscores the importance of recognizing anatomical variation and adapting standard cardiovascular workflows accordingly. As survival among patients with congenital heart disease continues to improve, generalists—including primary care and emergency physicians—are increasingly likely to encounter such patients in both chronic and acute care contexts. A unified mirror‐image approach may serve as a simple and transferable mental model, reducing cognitive load and facilitating rapid adaptation in time‐sensitive clinical situations. Sharing pragmatic, experience‐based adaptations—such as reversed limb‐lead placement, modified echocardiographic positioning, and tailored cardioversion pad configuration—may reduce uncertainty, support timely decision‐making, and improve patient safety outside specialized centers.

This report has several limitations. First, the present patient had complex congenital cardiac anomalies, including a single atrium and functional single ventricle physiology. Although these abnormalities had been diagnosed previously and were not the primary focus of the present report, they may influence the generalizability of the proposed approach. Because the mirror‐image workflow is based primarily on cardiac position rather than detailed intracardiac anatomy, the same conceptual approach may be applicable to patients with isolated dextrocardia; however, further validation across a broader spectrum of anatomical variants is required.

Second, the procedures were performed by primary care clinicians in consultation with an experienced cardiologist. Although the proposed workflow was designed as a simple and practical framework that may assist nonspecialist clinicians, its implementation should be supported by appropriate expertise when available. Additional visual guidance or supervised experience may be helpful before novice clinicians independently apply these modifications. Future studies should evaluate the reproducibility of this workflow among clinicians with varying levels of experience.

## 5 Conclusion

This case demonstrates that a unified mirror‐image approach can be successfully applied across electrocardiography, transthoracic echocardiography, and cardioversion in a patient with dextrocardia. By integrating these procedural adaptations into a unified workflow, clinicians may more easily recognize and manage dextrocardia in emergency and primary care settings. Further validation in patients with different anatomical variants and among clinicians with varying levels of experience is warranted.

## Author Contributions

**Rintaro Tamaruya:** writing – review and editing. **Junya Shimamoto:** writing – original draft.

## Funding

The authors have nothing to report.

## Ethics Statement

According to institutional policy, ethical committee approval is not required for single case reports. This case was conducted in accordance with the principles of the Declaration of Helsinki.

## Consent

Written informed consent was obtained from the patient for the publication of this case report and any accompanying images. All identifying information has been anonymized to protect patient privacy.

## Conflicts of Interest

The authors declare no conflicts of interest.

## Data Availability

The data that support the findings of this study are available on request from the corresponding author. The data are not publicly available due to privacy or ethical restrictions.
